# Supplementary material for: Revealing β-TrCP activity dynamics in live cells with a genetically encoded biosensor
Source: Nat Commun. 2022 Oct 26;13:6364. doi: 10.1038/s41467-022-33762-3 (PMC9606124; doi:10.1038/s41467-022-33762-3)
Supplement: Supplementary file 1 — SupplementaryInformation [file 41467_2022_33762_MOESM1_ESM.pdf]

SUPPLEMENTARY INFORMATION

Supplementary Figure 1
Supplementary Figure 2
Supplementary Figure 3
Supplementary Figure 4
Supplementary Figure 5
Supplementary Figure 6

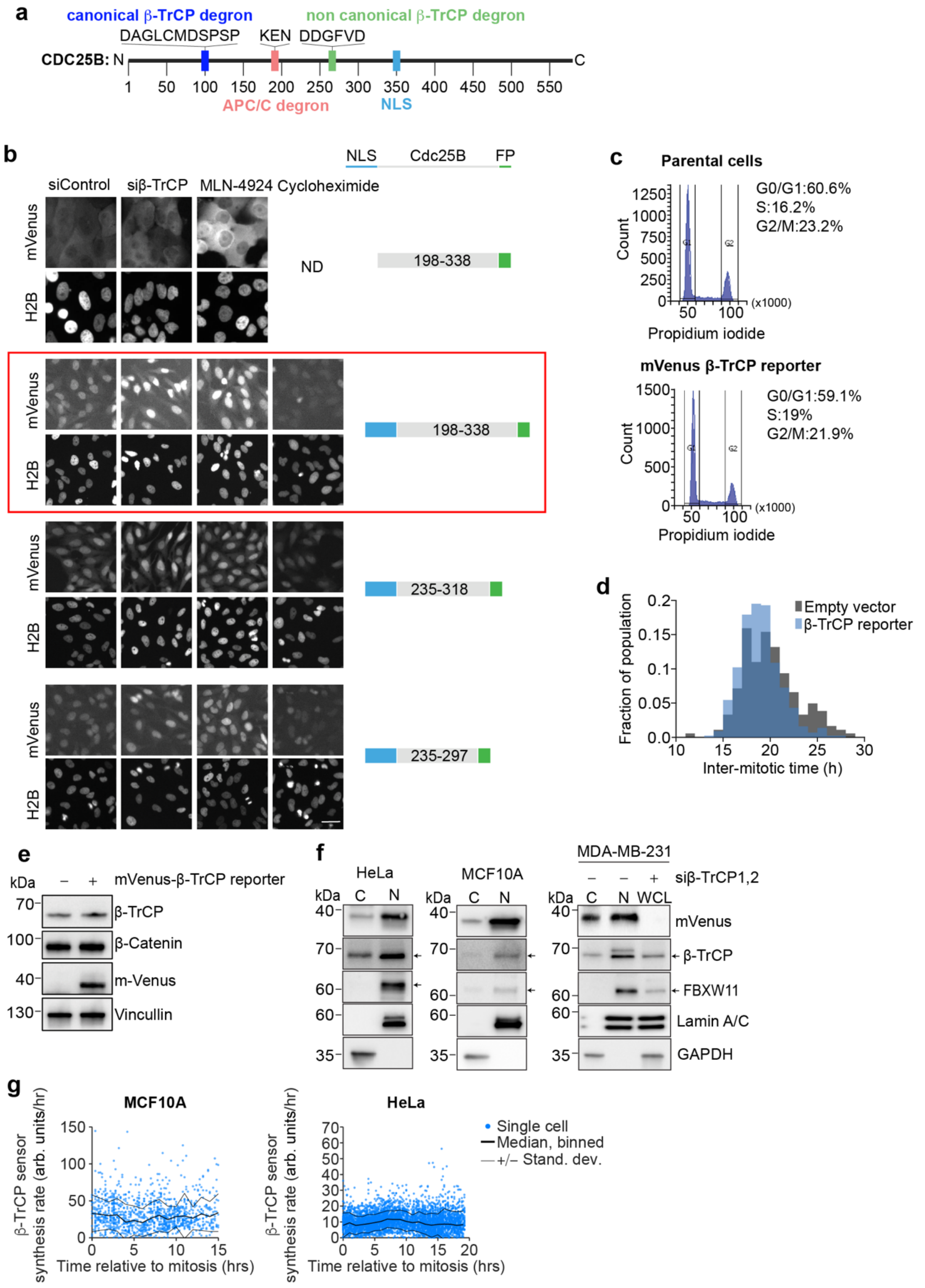

Supplementary Figure 1. The β-TrCP reporter does not alter cell cycle dynamics.

(a) Schematic showing human CDC25B sequence analysis and presence of both β-TrCP degrons and APC/C degrons. NLS, nuclear localization sequence.

(b) HeLa cells stably expressing H2B-mTurquoise and different human CDC25B-mVenus constructs were transfected with either control siRNA or β-TrCP1,2 siRNA for 48 h, or treated with MLN-4924 or Cycloheximide for 6 h before fixation. Red box indicates the chosen construct, now referred to as β-TrCP reporter. ND- Not done. Representative image from n=3 independent experiments. Scale bar is 10 µm.
(c) Parental HeLa cells and cells stably expressing the β-TrCP reporter were stained with propidium iodide and distribution of DNA content is measured via flow cytometry.
(d) Histogram of intermitotic times of parental HeLa cells and HeLa cells stably expressing the β-TrCP reporter. Intermitotic times were calculated following live-cell imaging and determining the time from one anaphase to the next anaphase.
(e) Parental HeLa cells and HeLa cells stably expressing the β-TrCP reporter were collected and whole cell lysates were immunoblotted for the indicated proteins. Representative blot of n=3 independent experiments.
(f) Asynchronously cycling HeLa, MCF10A, and MDA-MB-231 cells stably expressing the β-TrCP reporter were collected and fractionated to obtain cytoplasmic and nuclear protein pools followed by immunoblotting for the indicated proteins. An equal amount of whole cell lysate from MDA-MB-231 cells treated with siβ-TrCP1,2 (without expressing the β-TrCP reporter) was run alongside MDA-MB-231 fractionated samples to validate the localization and specificity of the β-TrCP1 and β-TrCP2 (FBXW11) antibodies. Representative blot from n=3 experiments. C, cytoplasmic; N, nuclear; WCL, whole cell lysate.
(g) Scatter plot of the time since mitosis plotted against the synthesis rate of the β-TrCP reporter in single MCF10A (left) or HeLa cells (right). Asynchronously cycling cells were imaged by live-cell imaging and tracked to establish the time since mitosis. Cells were then treated with MLN-4924 to inhibit β-TrCP activity. The slope of the resulting increase in β-TrCP reporter levels was measured and reported as the synthesis rate. Single-cell data was then binned to determine the median synthesis rate. Thick line reflects the median synthesis rate and thin lines represent the standard deviation.
Source data for all figure panels are provided as a Source Data file.

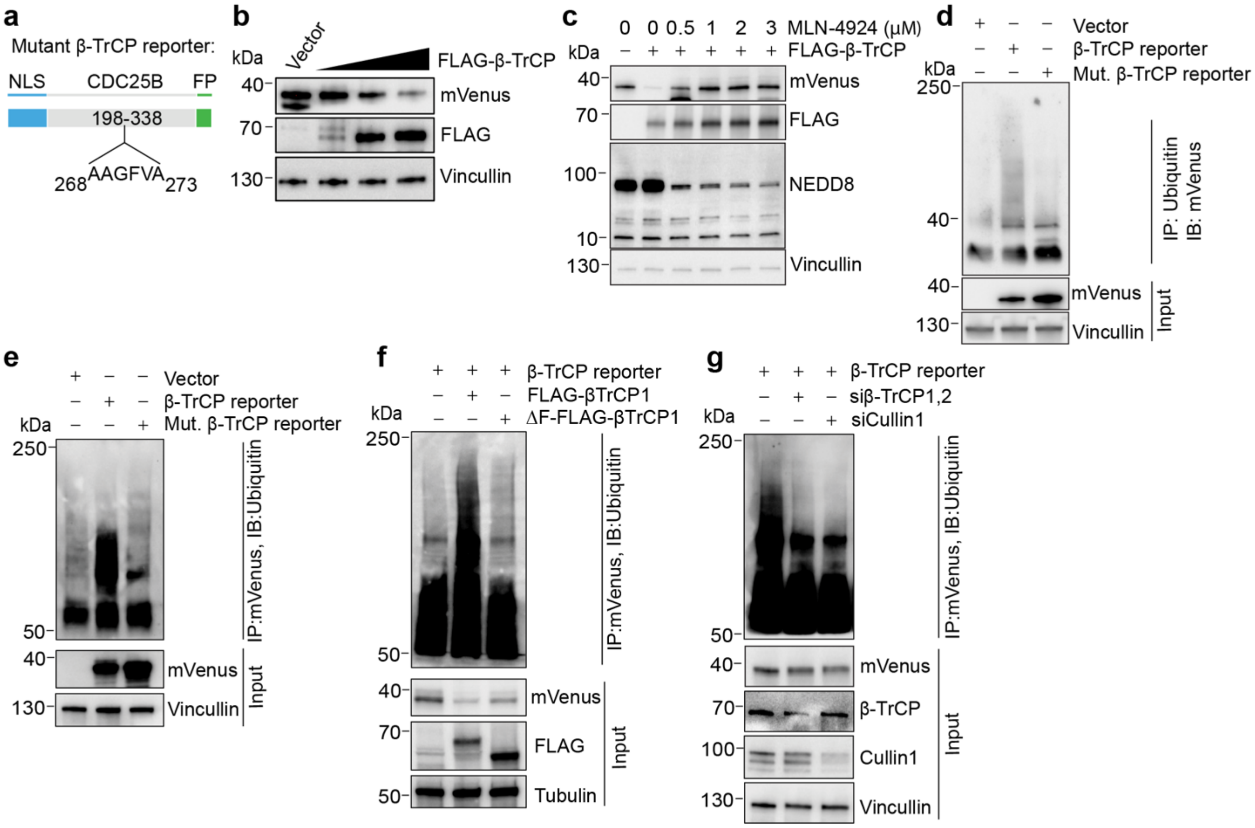

Supplementary Figure 2. The β-TrCP reporter degradation is β-TrCP-dependent.

(a) Schematic showing the mutant-β-TrCP reporter fused to mVenus. The non-canonical β-TrCP degron sequence of DDGFVD was mutated to AAGFVD.

(b) HeLa cells stably expressing the β-TrCP reporter were transfected with increasing amounts of β-TrCP. Whole cell lysates were immunoblotted for the indicated proteins. Representative blot of n=3 independent experiments.

(c) HEK-293T cells were transfected as indicated. Transfected cells were then treated with indicated dose of MLN-4924 for 4 h. Whole cell lysates were immunoblotted for the indicated proteins. Representative blot of n=3 independent experiments.

(d) HEK-293T cells were transfected as indicated. Transfected cells were then treated with MG132 (5 μM) for 6 h. Whole cell protein extracts were immunoprecipitated with anti-ubiquitin antibody and immunoprecipitates were immunoblotted for mVenus. High mass ladder indicates polyubiquitination. Representative blot of n=3 independent experiments.

(e) HEK-293T cells were transfected as indicated. Transfected cells were then treated with MG132 (5 μM) for 6 hrs. Whole cell protein extracts were immunoprecipitated with an mVenus antibody and immunoprecipitates were immunoblotted for ubiquitin. High mass ladder indicates polyubiquitination. Representative blot from n=3 experiments.

(f) HEK-293T cells were transfected with indicated constructs and then whole cell protein extracts were immunoprecipitated with an mVenus antibody. Immunoprecipitates were probed for ubiquitin antibody. Representative blot from n=3 experiments.

(g) HeLa cells stably expressing the mVenus-β-TrCP reporter, were transfected with the indicated siRNAs against β-TrCP1,2 or Cullin1. Cells were treated with MG132 (5 μM) for the 6 hrs before collection. Whole cell protein extracts were immunoprecipitated with an mVenus antibody and immunoblotted for ubiquitin antibody. Representative blot from n=2 experiments.

Source data for all figure panels are provided as a Source Data file.

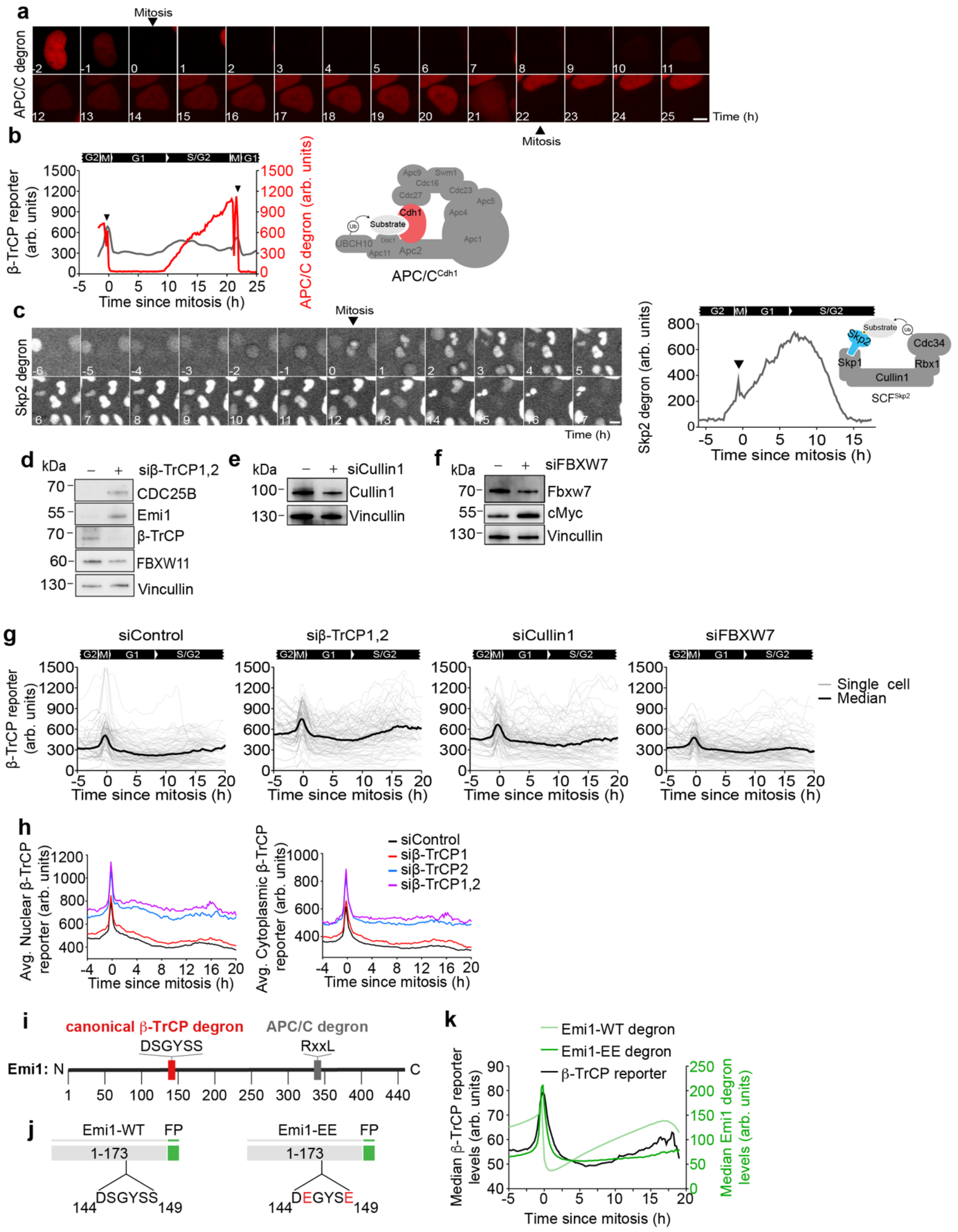

Supplementary Figure 3. In vivo validation of the β-TrCP reporter.

(a) Image montage of the same single-cell from Fig. 3a expressing the mCherry-APC/C ^{Cdh1} degron. Images were taken every 12 minutes and shown at one-hour intervals. The frame of mitosis is noted.

Scale bar is 10 μm.
(b) Levels of the β-TrCP reporter and the APC/C^{Cdh1} reporter from the cell in (a). The APC/C reporter levels were used to determine the timing of the G1/S transition. Right, schematic diagram depicting the APC/C^{Cdh1} complex.
(c) Image montage of a single representative HeLa cell expressing the Skp2 degron. Images were taken every 12 minutes and shown at one-hour intervals. The frame of mitosis is noted. Quantification of the Skp2 degron levels from the single cell shown. Inset is a schematic representation of SCF^{Skp2}. Scale bar is 10 μm.
(d) HeLa cells transfected with either control siRNA or β-TrCP1,2 siRNA Cells were collected after 48 h and whole cell lysates were immunoblotted for β-TrCP and two β-TrCP substrates, CDC25B and Emi1. Representative blot from n=3 experiments.
(e-f) HeLa cells transfected with either control siRNA, Cullin1 siRNA (e), or Fbxw7 siRNA (f). Cells were collected after 48 h and whole cell lysates were immunoblotted with the indicated antibody. Representative blots from n=3 experiments.
(g) Single-cell and median β-TrCP reporter levels in HeLa cells transfected with either control siRNA, β-TrCP1,2 siRNA, Cullin1 siRNA, or Fbxw7 siRNA. Single-cell traces were aligned to time of mitosis. N=88 (siControl), 100 (siβ-TrCP1,2), 100 (siCullin1), and 100 (siGBXW7) representative single cell traces. Median traces derived from N=88, 282, 135, and 227 cells respectively.
(h) Median nuclear and cytosplasmic β-TrCP reporter levels in HeLa cells treated with either siControl, siβ-TrCP1, β-TrCP 2, or β-TrCP1,2. Single-cell traces were computationally aligned to mitosis and the median trace was calculated.
(i) Schematic depicting the Emi1 protein sequence and the relative position of the β-TrCP degron and the APC/C degron.
(j) Schematic showing mVenus fused to fragments of either wild type (Emi1-WT) or a phospho-mimetic mutant of Emi1 (Emi1-EE). FP, fluorescent protein.
(k) Median β-TrCP reporter, Emi1-WT, and Emi1-EE levels in HeLa cells. HeLa cells stably co-expressing an mCherry-β-TrCP reporter and either an mVenus tagged Emi1-WT or an Emi1-EE were imaged. Single-cell traces were computationally aligned to mitosis and the median trace was calculated. Representative plot from n=2 experiments.
Source data for all figure panels are provided as a Source Data file.

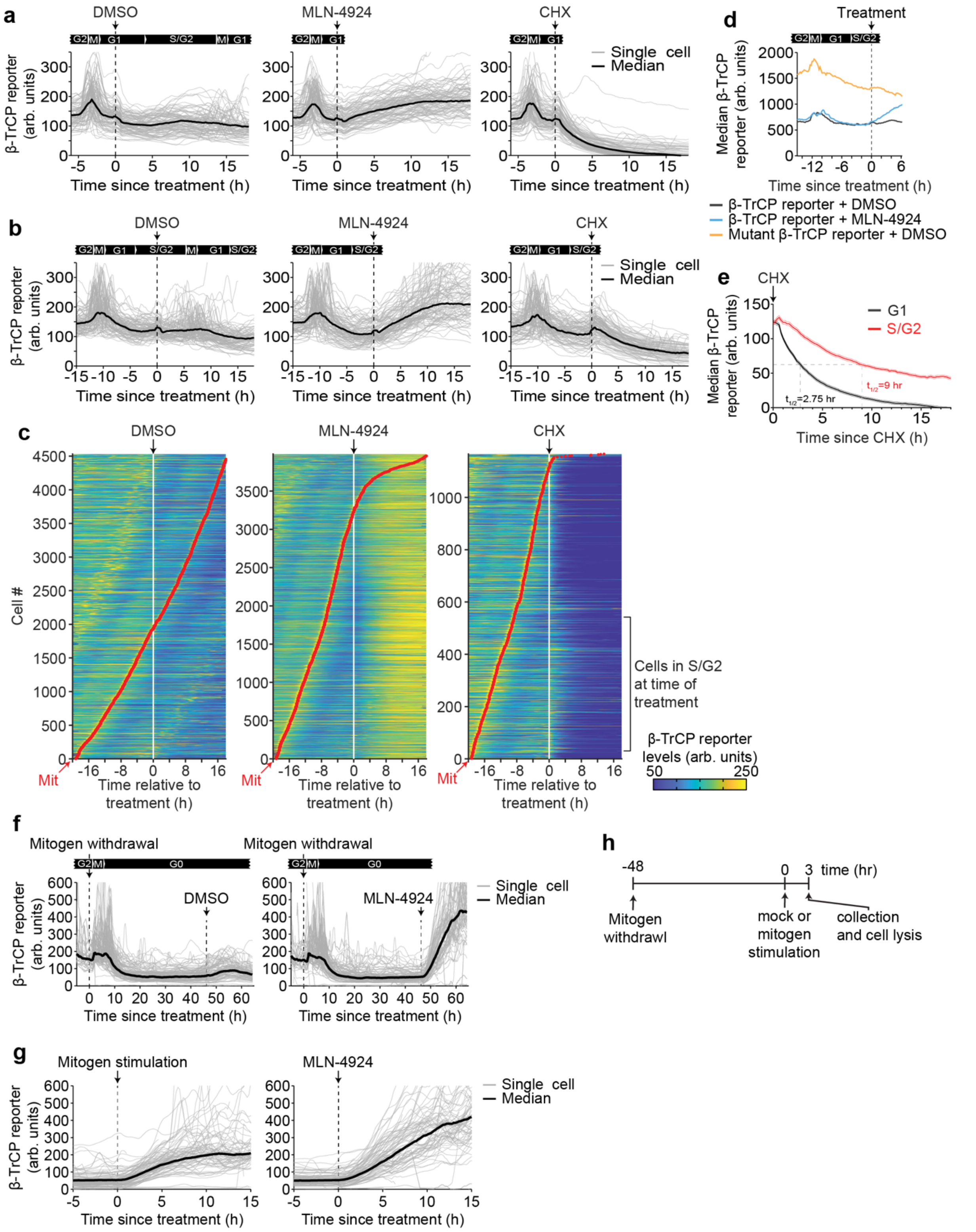

Supplementary Figure 4. β-TrCP reporter levels are controlled by degradation and synthesis.

(a-b) HeLa cells expressing the β-TrCP reporter were treated with either DMSO, MLN-4924 (3 µM) or cycloheximide (CHX; 100 µg/ml). Single cell traces (Gray lines) and median β-TrCP reporter levels are

shown. Only cells treated in G1 phase (a) or S/G2 phase (b) are shown. N=100 cells per condition. Representative traces from n=3 experiments.

(c) Heatmap of single cell β-TrCP reporter levels after treatment with DMSO, MLN-4924 (3 μM), or cycloheximide (CHX; 100 μg/ml) at the indicated by time. Mitosis is noted for each cell with a red dot. Representative heatmaps from n=3 experiments.

(d) Median reporter levels in HeLa cells stably expressing either the β-TrCP reporter or the mutant β-TrCP reporter. Blue line indicates cells expressing the β-TrCP reporter and then treated with MLN-4924 (3μM). Representative plot from n=2 experiments.

(e) Median β-TrCP reporter levels from cells treated with cycloheximide (CHX, 100 μg/mL) in either G1 or G2/M phase. Shaded region indicates SEM. Representative figure from n=3 experiments.

(f) Median (Black) and single cell traces (Gray) of β-TrCP reporter levels in MCF-10A cells. Cells were washed to remove all mitogens at the indicated times and then subsequently treated with either DMSO or MLN-4924 (3μM). N=100 cells per condition.

(g) Median (Black) and single cell traces (Gray) of β-TrCP reporter levels in MCF-10A cells. Cells were first serum starved for 48 h to induce quiescence. At the indicated times, cells were treated with either full growth media (Mitogen stimulation) or mitogen-free media combined with MLN-4924 (3μM). N=100 cells per condition.

(h) Experimental design for Fig. 3l. MCF10A cells were first mitogen-starved for 48 h to induce quiescence. Cells were then stimulated with either complete growth media (mitogen stimulated) or mitogen starvation media (mock stimulated). After 3 h, cells were collected, lysed, and processed for western blotting.

Source data for all figure panels are provided as a Source Data file.

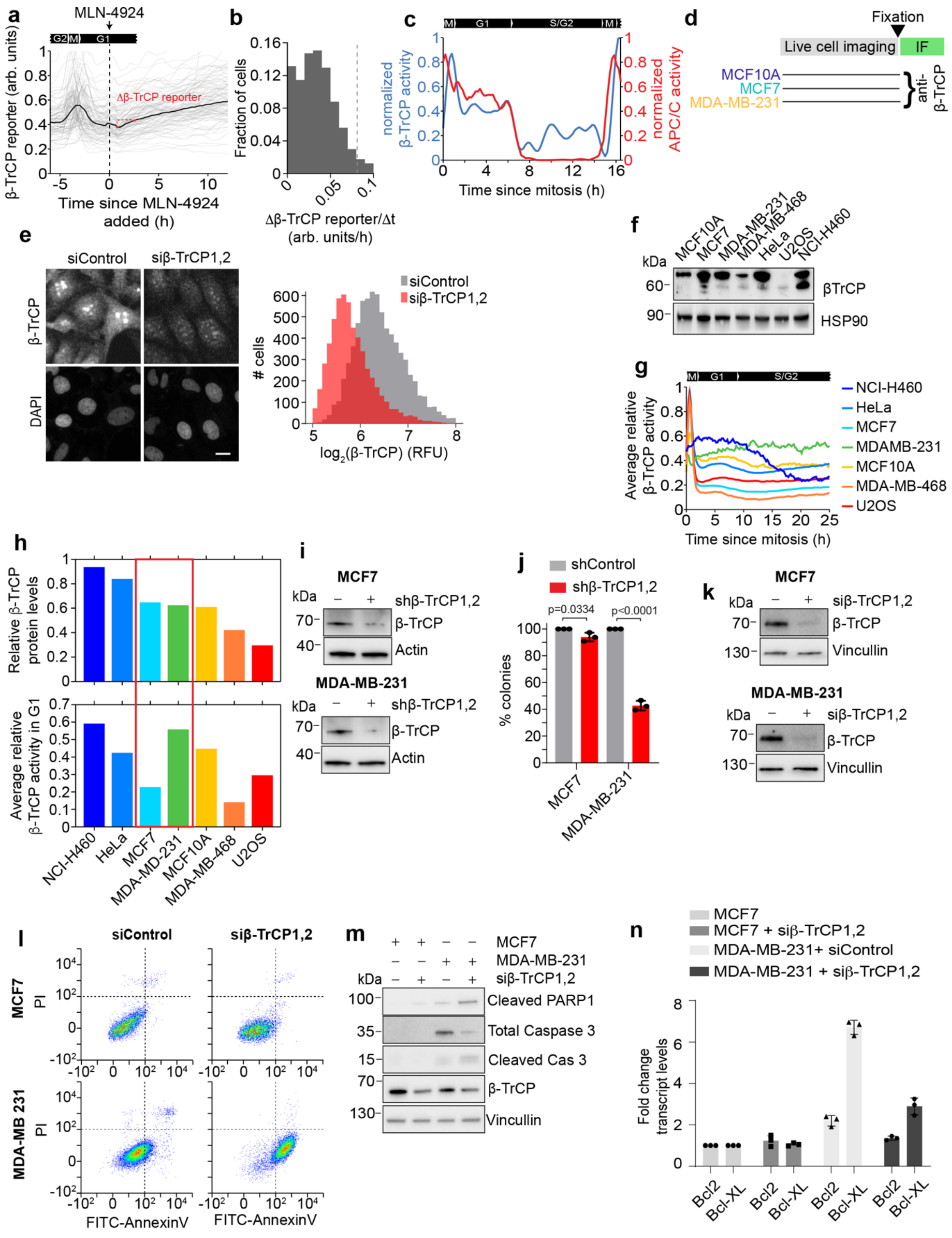

Supplementary Figure 5. β-TrCP activity does not correlate with β-TrCP protein levels.

(a) Single-cell levels of β-TrCP reporter in HeLa cells treated with MLN-4924 (3μM) at the indicated time. To estimate the rate of β-TrCP reporter accumulation, or k-synthesis, the change in β-TrCP reporter

levels was calculated over a 2 h time window 1-2 h after treatment with MLN-4924.

(b) Histogram of the β-TrCP reporter synthesis rate from single cells in (a). The 90th percentile synthesis rate was used to convert β-TrCP reporter levels into β-TrCP activity.

(c) Normalized activity of β-TrCP (Blue) and APC/C (Red) activity from a single cell (see Fig. 4a).

(d) Schematic showing outline of experiment performed in (e). Cells were pre-imaged to measure β-TrCP activity at the single cell level. Cells were then fixed and immunostained with a β-TrCP antibody. Cells were then registered back to the last image taken from time lapse imaging to link the live-cell images to the fixed cell images.

(e) MCF7 cells were transfected with either control siRNA or β-TrCP1,2 siRNA for 48 h before fixation and stained for anti- β-TrCP antibody. Representative images from n=3 experiments.
Histograms depicting b-TrCP reporter levels in single-cells. N= 7,307 (sicontrol) and 5,602 (siβ-TrCP1,2) cells. Scale bar is 10 µm.

(f) Immunoblot showing the expression of β-TrCP in different cell lines. Exponentially growing Cells were collected, lysed, and whole cell lysates were probed for indicated proteins. Representative blot of n=2 independent experiments.

(g) Median β-TrCP activity in the indicated cell lines.

(h) Bar graph showing relative β-TrCP protein levels (upper panel) and relative β-TrCP activity (lower panel) in the indicated cell line. Cell-lines were ordered based on β-TrCP protein levels. Note the similar β-TrCP protein levels in MCF7 and MDA-MB-231 cell lines but differential β-TrCP activity. n=2 experiments.

(i) MCF7 cells (top) or MDA-MB-231 cells (bottom) were transduced with either control shRNA or β-TrCP shRNA for 60 h. Whole cell lysates were immunoblotted for indicated proteins. Representative blots for n=3 independent experiments.

(j) MCF7 and MDA-MB-231 cells stably expressing sh-β-TrCP1,2 were allowed to form colonies (Fig. 4f), fixed, stained, counted, and represented as percent control shRNA. Error bars represent SEM from n=3 experiments. P values were calculated using one-sided student's t test.

(k) MCF7 cells (top) or MDA-MB-231 cells (bottom) were transfected with either control siRNA or β-TrCP1,2 siRNA for 48 h. Whole cell lysates were immunoblotted for indicated proteins. Representative blots for n=3 independent experiments.

(l) Annexin-V5 and propidium iodide (PI) staining of MCF7 and MDA-MB-231 cells transfected with either control siRNA or β-TrCP1,2 siRNA. Representative plot from n=5 experiments.

(m) Immunoblot showing the expression of Cleaved PARP1, total and cleaved caspase 3 in MCF7 and MDA-MB-231 cells treated with β-TrCP1,2 siRNA. Exponentially growing Cells were collected, lysed,

and whole cell lysates were probed for indicated proteins. Representative blot of n=3 independent experiments.

(n) Relative mean transcript levels of Bcl2 and Bcl-XL in MCF7 and MDA-MB-231 cells. Cells were transfected with either control or β-TrCP1,2 siRNA. Error bars represent SD from n=3 experiments.

Source data for all figure panels are provided as a Source Data file.

a

β-TrCP activity	Compound Name	Primary MOA	Library	AC50 (μM)	Efficacy	CC-v2
Inhibiting	MG-132	Proteasome inhibitor	MIPE	0.700	89.30	1.1
Inhibiting	Bortezomib	Proteasome inhibitor	NPC/MIPE	0.025	94.59	1.1
Inhibiting	Ixazomib citrate	Proteasome inhibitor	NPC/MIPE	0.176	98.42	1.1
Inhibiting	Carfilzomib	Proteasome inhibitor	NPC/MIPE	0.028	90.50	1.1
Inhibiting	Marizomib	Proteasome inhibitor	MIPE	0.011	84.93	1.1
Inhibiting	MLN-4924	NAE inhibitor	MIPE	0.018	60.79	1.2
Inhibiting	Oprozomib	Proteasome inhibitor	MIPE	0.624	90.15	1.2
Inhibiting	MG-115	Proteasome inhibitor	MIPE	0.785	72.24	1.2
Inhibiting	Delanzomib	Proteasome inhibitor	MIPE	0.056	69.45	1.2
Inhibiting	MLN-7243	Ubiquitin-Activating Enzyme E1 inhibitor	MIPE	0.221	70.09	1.2
Inhibiting	MLN-9708	Proteasome subunit beta type-5 inhibitor	NPC	0.124	78.58	1.2
Activating	Cycloheximide	Protein translation inhibitor	MIPE	0.351	-58.69	-1.2
Activating	Omacetaxine mepesuccinate	Protein translation inhibitor	NPC/MIPE	0.035	-59.56	-1.2
Activating	Sunitinib malate	VEGFR/PDGFR inhibitor	NPC	4.415	-48.19	-2.2
Activating	CT-99021	GSK-3 inhibitor	MIPE	3.507	-105.90	-1.1
Activating	ASP-3026	ALK inhibitor	MIPE	8.809	-57.93	-2.2
Activating	GSK-615	PI3Kalpha inhibitor	MIPE	3.126	-74.23	-2.2
Activating	PP-121	PDGFR inhibitor	MIPE	1.244	-60.77	-1.1
Activating	HMSL10077	BTK inhibitor	MIPE	3.507	-55.85	-1.1
Activating	Rociletinib	EGFR inhibitor	MIPE	7.386	-65.06	-1.2
Activating	Avitinib	EGFR inhibitor	MIPE	4.660	-52.81	-1.2
Activating	VLX-1570	USP14 inhibitor	MIPE	3.017	-56.07	-1.2
Activating	Pemafibrate	PPARα inhibitor	MIPE	11.706	-98.16	-1.2
Activating	SGI-7079	AXL inhibitor	MIPE	1.473	-39.52	-1.2
Activating	ASP-3026	ALK inhibitor	MIPE	5.866	-52.79	-1.2
Activating	CPI-4203	KDM5 demethylase inhibitor	MIPE	1.043	-94.66	-1.1

b

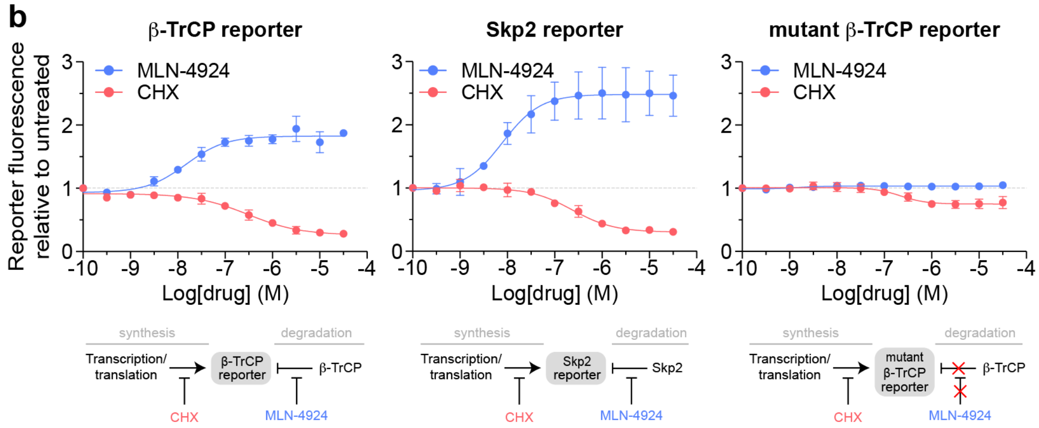

c

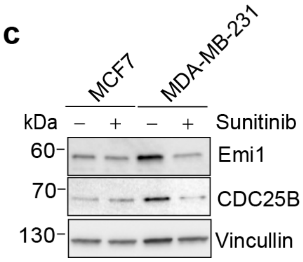

Supplementary Figure 6. Counter-screen and follow-up validation for HTS hits.

(a) Table summarizing validated hits from the combined compound collection screening (some represented in both collections, as indicated) and calculated IC50 (μM), Curve Class (CC-v2), and

Efficacy based on 11pt. dose response follow-up testing. Compounds are categorized as either β-TrCP inhibitors or activators based on their response. MOA, mechanism of action.
(b) MDA-MB-231 cells expressing the indicated reporter fused to mVenus were treated with increasing doses of either MLN-4924 or cycloheximide (CHX). Cells were imaged 6 hours after drug treatment. Data represents the median single-cell fluorescence intensity, normalized to untreated cells. Error bars represent SEM from n=2 experiments. Note that MLN-4924 and CHX have similar effects on the β-TrCP reporter and the Skp2 reporter, while only cycloheximide effected the mutant β-TrCP reporter. These counter-screen tools allow us to identify compounds with non-specific effects on cullin-mediated ubiquitination, proteasomal degradation, or non-ubiquitin related processes such as transcription or translation. (See below schematics)
(c) Exponentially growing MCF7 or MDA-MB-231 cells were treated with either DMSO or Sunitinib (3 μM). Whole cell lysates were resolved in SDS-PAGE and immunoblotted for the indicated proteins. Representative blot of n=3 independent experiments.
Source data for all figure panels are provided as a Source Data file.
