# Supplementary material for: Revealing β-TrCP activity dynamics in live cells with a genetically encoded biosensor
Source: Nat Commun. 2022 Oct 26;13:6364. doi: 10.1038/s41467-022-33762-3 (PMC9606124; doi:10.1038/s41467-022-33762-3)
Supplement: Supplementary file 3 — Reporting Summary [file 41467_2022_33762_MOESM3_ESM.pdf]

nature portfolio

Corresponding author(s): Steven D Cappell

Last updated by author(s): 2022/09/21

Reporting Summary

Nature Portfolio wishes to improve the reproducibility of the work that we publish. This form provides structure for consistency and transparency in reporting. For further information on Nature Portfolio policies, see our Editorial Policies and the Editorial Policy Checklist.

Statistics

For all statistical analyses, confirm that the following items are present in the figure legend, table legend, main text, or Methods section.

n/a	Confirmed	
☐	☒	The exact sample size (*n*) for each experimental group/condition, given as a discrete number and unit of measurement
☐	☒	A statement on whether measurements were taken from distinct samples or whether the same sample was measured repeatedly
☐	☒	The statistical test(s) used AND whether they are one- or two-sided<br>*Only common tests should be described solely by name; describe more complex techniques in the Methods section.*
☒	☐	A description of all covariates tested
☒	☐	A description of any assumptions or corrections, such as tests of normality and adjustment for multiple comparisons
☐	☒	A full description of the statistical parameters including central tendency (e.g. means) or other basic estimates (e.g. regression coefficient) AND variation (e.g. standard deviation) or associated estimates of uncertainty (e.g. confidence intervals)
☐	☒	For null hypothesis testing, the test statistic (e.g. *F*, *t*, *r*) with confidence intervals, effect sizes, degrees of freedom and *P* value noted<br>*Give P values as exact values whenever suitable.*
☒	☐	For Bayesian analysis, information on the choice of priors and Markov chain Monte Carlo settings
☒	☐	For hierarchical and complex designs, identification of the appropriate level for tests and full reporting of outcomes
☒	☐	Estimates of effect sizes (e.g. Cohen's *d*, Pearson's *r*), indicating how they were calculated

Our web collection on statistics for biologists contains articles on many of the points above.

Software and code

Policy information about availability of computer code

Data collection	NIS Elements (v5.11.00), Alpha View (v3.5.0.927), FlowJo V10
Data analysis	MATLAB (vR2020b). Automated image analysis was performed using custom MATLAB scripts as described in Cappell, S.D. et al Cell 166, 167-180 (2016).

For manuscripts utilizing custom algorithms or software that are central to the research but not yet described in published literature, software must be made available to editors and reviewers. We strongly encourage code deposition in a community repository (e.g. GitHub). See the Nature Portfolio guidelines for submitting code & software for further information.

Data

Policy information about availability of data

All manuscripts must include a data availability statement. This statement should provide the following information, where applicable:

- Accession codes, unique identifiers, or web links for publicly available datasets
- A description of any restrictions on data availability
- For clinical datasets or third party data, please ensure that the statement adheres to our policy

All data is available in the Source Data file. The datasets generated during and/or analyzed during the current study are also available from the corresponding author on reasonable request.

Field-specific reporting

Please select the one below that is the best fit for your research. If you are not sure, read the appropriate sections before making your selection.

☒ Life sciences ☐ Behavioural & social sciences ☐ Ecological, evolutionary & environmental sciences

For a reference copy of the document with all sections, see nature.com/documents/nr-reporting-summary-flat.pdf

Life sciences study design

All studies must disclose on these points even when the disclosure is negative.

Sample size	No statistical method was used to pre-determine sample size. The sample size was chosen to be at least 3 across the manuscript to enable statistical analysis.
Data exclusions	No data was excluded form the experiments.
Replication	All experiments in which p-values are present have been carried out with at least 3 replicates. All experiments were independently reproduced at least twice.
Randomization	Representative single-cell traces where chosen at random from the population.
Blinding	Blinding was not relevant to this study. Image acquisition and analysis was conducted using automated scripts which are not subject to experimental bias. For western blots, blinding is not possible because samples need to be loaded in a particular order.

Reporting for specific materials, systems and methods

We require information from authors about some types of materials, experimental systems and methods used in many studies. Here, indicate whether each material, system or method listed is relevant to your study. If you are not sure if a list item applies to your research, read the appropriate section before selecting a response.

Materials & experimental systems

n/a	Involved in the study	
☐	☒	Antibodies
☐	☒	Eukaryotic cell lines
☒	☐	Palaeontology and archaeology
☒	☐	Animals and other organisms
☒	☐	Human research participants
☒	☐	Clinical data
☒	☐	Dual use research of concern

Methods

n/a	Involved in the study	
☒	☐	ChIP-seq
☐	☒	Flow cytometry
☒	☐	MRI-based neuroimaging

Antibodies

Antibodies used

mVenus (MyBioSource, MBS448126, 1:1000)
beta-TrCP (Abcam, ab71753, 1:800)
beta-TrCP (SCBT, sc390629, used for IPs, 2ug)
beta-TrCP (CST, 4349, 1:250)
beta-catenin (BD Bioscience, BD-610153, 1:2500)
vinculin (sigma, V9131, 1:5000)
FBXW7 (Abcam, ab109617, 1:800)
FLAG (Sigma, Clone M2, F3165, 1:2000)
Ubiquitin (SCBT, Sc8017, 1:800)
Cullin1 (SCBT, sc17775, 1:800)
Skp1 (SCBT, sc5281, 1:700)
CDC25B (CST, 9525B, 1:1000)
Emi1 (SCBT, sc365212, 1:800)
HSP90 (CST, 4877, 1:1000)
Bcl-XL (CST, 4877, 1:1000)
Bcl2 (CST, 3498, 1:1000)
p65 (CST, 8242, 1:1000)
IKBα (CST, 4812, 1:1000)
caspase 3 (CST, 14220, 1:1000)
Cullin 1 (CST, 4995, 1:1000)

cleaved PARP (CST, 5625, 1:1000)
NEDD8 (CST, 2745, 1:1000)
FBXO31 (Bethyl, A302-047A, 1:800)
mouse IgG (SCBT, sc2025, used for IPs, 2ug)
DYRK1A (SCBT, sc100376, 1:800)
PFKFB3 (MyBioSource, MBS9604769, 1:1000)
FBXW5 (MyBioSource, MBS9611762, 1:1000)
FBXO25 (MyBioSource, MBS3017705, 1:1000)
FBXW11 (Thermo scientific, PA5-109715, 1:1000)
cMyc (Abcam, ab32072, 1:800)
Beta-actin (Abcam, ab6276, 1:5000)
Anti-rabbit IgG, HRP-linked antibody (CST-7074, 1:10000)
anti-mouse IgG, HRP linked antibody (CST-7076, 1:10000)
mouse anti-goat IgF, HRP secondary antibody (SCBT, sc2354, 1:15000)

Validation

All the antibodies used in this study are commercially available and extensively validated by the company, us, or others. Validation data is available in each of these company's website. In addition to the validation data of B-TrCP antibody in its manufacturer's website (Abcam, WB; CST, IF), we have confirmed the specificity of the B-TrCP antibody in our lab using western blot (Supplementary Fig. 1f, 3d, 5i and k) and immunofluoresence (Supplementary Fig. 5e) in B-TrCP knockdown human (HeLa, MCF10A, MCF7, and MDA-MB-231) cell lines.

Eukaryotic cell lines

Policy information about cell lines

Cell line source(s)

MCF10A (ATCC: CRL-10317)
HeLa (ATCC: CRM-CCL-2)
NCI-H460 (ATCC: HTB-177)
HEK293T (gift from Dr. Tobias Meyer's Laboratory at Weil Cornell Medical School, ATCC: CRL3216)
MCF7 (gift from Dr. Jing Huang's Laboratory at the National Cancer Institute, ATCC: HTB-22)
MDA-MB-231 (gift from Dr. Stuart Yuspa's Laboratory at the National Cancer Institute, ATCC: CRM-HTB-26)
MDA-MB-468 (gift from Dr. Stuart Yuspa's Laboratory at the National Cancer Institute, ATCC: HTB-132)
U2OS (gift form Dr. Li Yang's Laboratory at the National Cancer Institute, ATCC: HTB-96)

Authentication

Cell lines purchased from ATCC were not further authenticated. MCF7 cells were authenticated by short terminal repeat (STR) analysis peformed by the Huang Lab.

Mycoplasma contamination

Cells used in all experiments were routinely tested for mycoplasma continimation and only mycoplasma-negative cells were used in experiments

Commonly misidentified lines
(See ICLAC register)

No commonly misidentified cell lines were used in the study

Flow Cytometry

Plots

Confirm that:

- [x] The axis labels state the marker and fluorochrome used (e.g. CD4-FITC).
- [x] The axis scales are clearly visible. Include numbers along axes only for bottom left plot of group (a 'group' is an analysis of identical markers).
- [x] All plots are contour plots with outliers or pseudocolor plots.
- [x] A numerical value for number of cells or percentage (with statistics) is provided.

Methodology

Sample preparation

Harvested HeLa (supplementary Fig. 1c) cells or MCF7 and MDA-MD-231 (Supplementary Fig. 5l) cells washed in PBS, after which 1x106 cells were resuspended and stained using Dead Cell Apoptosis Kit with Annexin V FITC and PI (V13242, Thermo Fisher) according to manufacturer's instructions.

Instrument

BD FACS Calibur (Supplementary Fig. 1c) and BD FACSCanto II (Fig. 4g and Supplementary Fig. 5l)

Software

BD FACSDiVa Software was used for data collection, FLowJO v10 was used for data analysis.

Cell population abundance

For each sample, 20,000 cells were loaded onto the cytometer and a minimum of 10,000 were available post-sort for data collection and analysis.

Gating strategy	FSC/SCC gate were used to select all cells, then FSC-A and SCC-A gates were used to select singlet cells.

☒ Tick this box to confirm that a figure exemplifying the gating strategy is provided in the Supplementary Information.
